# Latent diffusion augmentation enhances deep learning analysis of neuro-morphology in limbal stem cell deficiency

**DOI:** 10.3389/fmed.2023.1270570

**Published:** 2023-10-16

**Authors:** David Gibson, Thai Tran, Vidhur Raveendran, Clémence Bonnet, Nathan Siu, Micah Vinet, Theo Stoddard-Bennett, Corey Arnold, Sophie X. Deng, William Speier

**Affiliations:** ^1^Medical Informatics Home Area, Graduate Programs in Bioscience, University of California, Los Angeles, Los Angeles, CA, United States; ^2^Department of Bioengineering, University of California, Los Angeles, Los Angeles, CA, United States; ^3^Ophthalmology Department, Cochin Hospital and Paris Cité University, AP-HP, Paris, France; ^4^Stein Eye Institute, University of California, Los Angeles, Los Angeles, CA, United States; ^5^Computational Diagnostics Lab, University of California, Los Angeles, Los Angeles, CA, United States; ^6^David Geffen School of Medicine, University of California, Los Angeles, Los Angeles, CA, United States; ^7^Molecular Biology Institute, University of California, Los Angeles, Los Angeles, CA, United States

**Keywords:** deep learning, ophthalmology, machine learning, limbal stem cell deficiency, *in vivo* confocal microscopy

## Abstract

**Introduction:**

Limbal Stem Cell Deficiency (LSCD) is a blinding corneal disease characterized by the loss of function or deficiency in adult stem cells located at the junction between the cornea and the sclera (i.e., the limbus), namely the limbal stem cells (LSCs). Recent advances in *in vivo* imaging technology have improved disease diagnosis and staging to quantify several biomarkers of *in vivo* LSC function including epithelial thickness measured by anterior segment optical coherence tomography, and basal epithelial cell density and subbasal nerve plexus by *in vivo* confocal microscopy. A decrease in central corneal sub-basal nerve density and nerve fiber and branching number has been shown to correlate with the severity of the disease in parallel with increased nerve tortuosity. Yet, image acquisition and manual quantification require a high level of expertise and are time-consuming. Manual quantification presents inevitable interobserver variability.

**Methods:**

The current study employs a novel deep learning approach to classify neuron morphology in various LSCD stages and healthy controls, by integrating images created through latent diffusion augmentation. The proposed model, a residual U-Net, is based in part on the InceptionResNetV2 transfer learning model.

**Results:**

Deep learning was able to determine fiber number, branching, and fiber length with high accuracy (R2 of 0.63, 0.63, and 0.80, respectively). The model trained on images generated through latent diffusion on average outperformed the same model when trained on solely original images. The model was also able to detect LSCD with an AUC of 0.867, which showed slightly higher performance compared to classification using manually assessed metrics.

**Discussion:**

The results suggest that utilizing latent diffusion to supplement training data may be effective in bolstering model performance. The results of the model emphasize the ability as well as the shortcomings of this novel deep learning approach to predict various nerve morphology metrics as well as LSCD disease severity.

## Introduction

Limbal stem cells (LSCs) are adult stem cells located at the junction between the cornea and the sclera which are responsible for continuous corneal epithelial renewal (1). Limbal stem cell deficiency (LSCD) is a potentially blinding corneal disease caused by a loss of function functional LSCs (2). This condition presents with debilitating symptoms including photophobia, burning, irritation, and loss of vision potentially leading to blindness. Without LSCs, conjunctival epithelial cells invade the corneal surface leading to decreased vision as a result of conjunctivalization of the cornea (3). Recent guidelines have clarified the disease definition, diagnosis, staging, and management of LSCD (2, 4).

As clinical presentation does not always correlate with the level of LSCD or *in vivo* LSC function (5, 6), it is recommended to perform additional diagnostic tests including *in vivo* imaging such as anterior segment optical coherence tomography (AS-OCT) and *in vivo* laser scanning confocal microscopy (ICVM) to evaluate *in vivo* biomarkers of the disease (2, 7). A composite score correlating with disease severity can then be generated by combining these biomarkers (7). One of the biomarkers is the central corneal sub-basal nerve density (SND) (8, 9). A decrease in SND correlates with the severity of LSCD. Other nerve parameters correlating with the severity of LSCD include central corneal sub-basal nerve branching number, fiber number, and fiber tortuosity (7, 8). Quantification of these nerve parameters requires highly trained personnel to manually annotate images, is time-consuming, and is open to interrater variability.

There have been many other attempts at automating the process of neuro-morphological classification outside of the cornea. Most of the current literature focuses on the segmentation of neuro-images and classifying neurons through the use of various deep learning algorithms (10, 11). While many approaches use convolutional neural networks (CNN), recent benchmarking efforts have revealed that linear discriminant analysis (LDA) can serve as a promising discriminatory classifier (10). Prior research in other domains of ophthalmology has used deep learning models to aid in the diagnosis of diabetic neuropathy and fungal keratitis using IVCM images (12–14). Diagnostic challenges can be remedied by integrating recent advances in computational approaches into the current clinical workflow. This approach can increase diagnosis precision and reduce time-to-treatment and clinician burden.

To address these challenges, our morphological classifier automates the process of diagnosing LSCD using nerve morphology features from ICVM images. Total corneal nerve fiber length, corneal nerve fiber density, corneal nerve branch density, and tortuosity coefficient are among the nerve morphology biomarkers used for disease staging clinically (7). These biomarkers have been shown to correlate significantly with LSCD as well as other biomarkers such as basal cell density (8, 9). Examining these biomarkers will elucidate how these quantifiable morphology features relate to LSCD disease progression. We employed deep learning to classify neuron morphology. To maximize the effectiveness of these models we developed a novel pre-processing pipeline for use prior to training and testing. To overcome potential hurdles with the size of our data set we employed random sampling with replacement, as well as image augmentation and enhancement. Stable diffusion (SD) is a latent diffusion model which is generally used as a text-to-image model (15). Deep learning requires large datasets and diffusion models present an opportunity for more robust data augmentation beyond typical image transformations like rotations and flips (16). A number of recent studies have explored diffusion models for specific tasks in medical imaging, including synthesizing magnetic resonance imaging and computed tomography volume scans (17). To date, no other work in neuro-morphological classification has incorporated artificially generated images into training datasets using SD. The current study is the first to demonstrate the use of this approach in subbasal nerve analysis.

## Materials and methods

### Dataset

Appropriate consent was obtained from study subjects in accordance to IRB protocol (UCLA IRB #10-001601). The study was compliant with HIPAA regulations and adhered to the Declaration of Helsinki.

**Figure 1:**
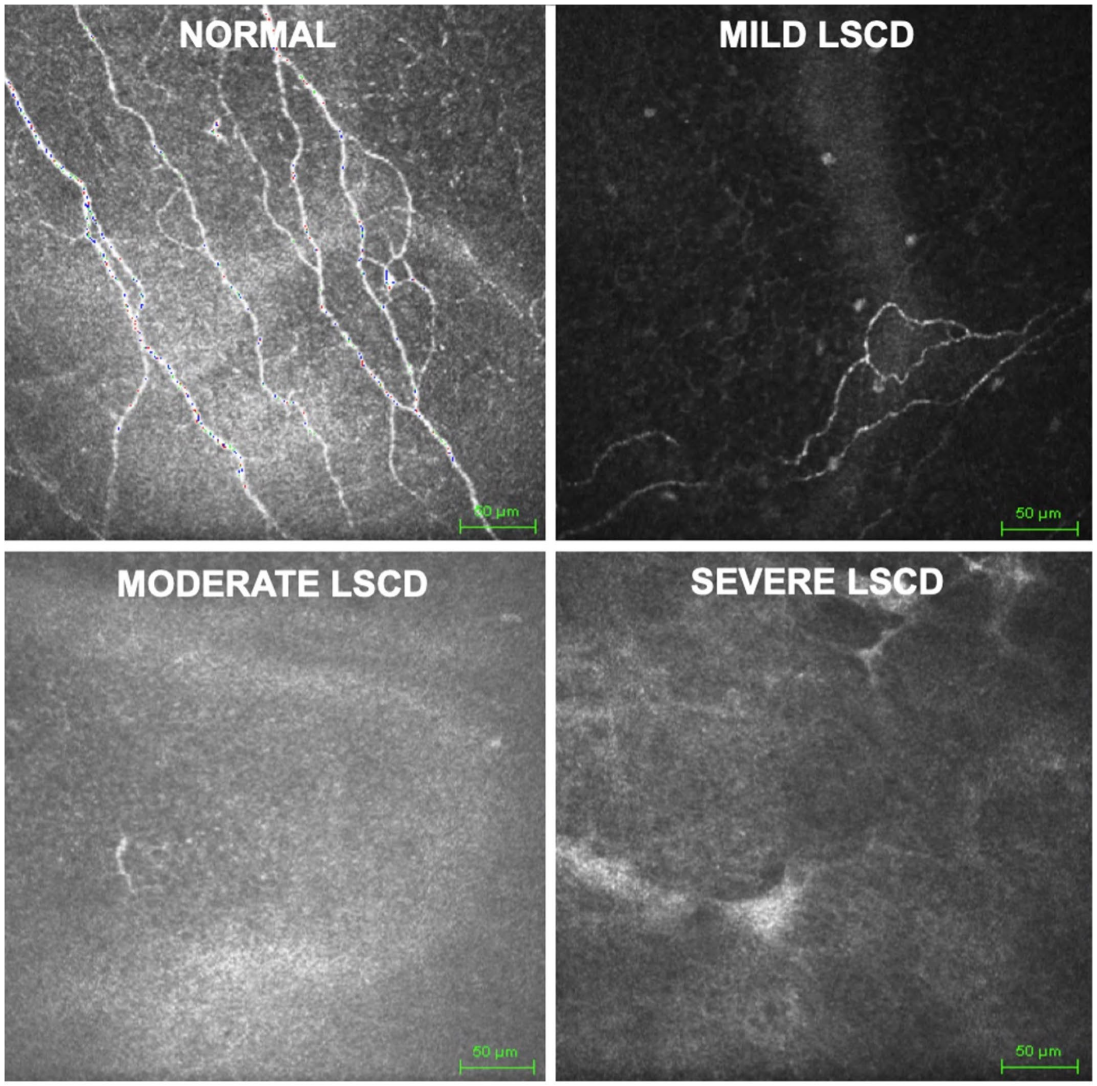
Grayscale *in vivo* confocal microscopy images of central cornea used as data source. Classification (control, mild, moderate, severe) based on presence, density, branching, and tortuosity of visible nerves.

LSCD diagnosis was based on a comprehensive examination including history, slit lamp examination, and fluorescein-staining pattern, and confirmed in all cases by IVCM and/or AS-OCT and impression cytology (2). The control group included patients without any ocular or systemic morbidities and a normal ocular examination. The stage of LSCD was classified as mild (2–4 points), moderate (5–7 points), or severe (8–10 points) based on a clinical scoring system previously published (18). IVCM volume scans of the central cornea were obtained from 133 patients clinically presenting with LSCD and 54 healthy controls. Of the 187 volume scans, 62 were Mild (Class I), 55 were Moderate (Class II), and 16 were Severe (Class III). Figure 1 shows an example case of each severity. In total, 641 individual scans were obtained. IVCM scans were performed using HRT III (Heidelberg Engineering GmBH, Germany) with the Rostock cornea module at the Stein Eye Institute, University of California, Los Angeles. A minimum of three high-quality Z-scans were acquired in the central cornea from the superficial epithelium down to the anterior stroma (40 scans of 400 μm × 400 μm, one every 2 microns, representing 8-bit grayscale 384 × 384 pixels). For each eye, up to 4 individual scans from the volume were identified by a senior cornea specialist (CB) as clinically relevant for nerve morphology identification and quantified by two trained readers for disease severity, fiber number, fiber length, branch number, and nerve tortuosity. The average of the provided labels by the two readers was used as ground truth labels for the quantification task.

### Image preprocessing

**Figure 2:**
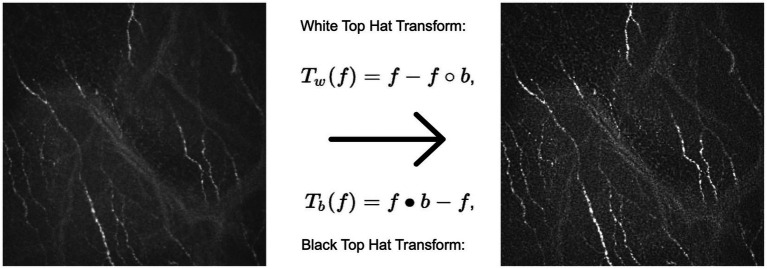
Example of an original image (left) subject to the Top Hat and Black Hat transformation, resulting in a contrast enhanced image (right) with highlighted neurons compared to the original.

To enhance the visibility of the structures of interest, we applied a combination of Top Hat and Black Hat transformations. The Top Hat transform is designed to find bright objects on a dark background, while the Black Hat transform does the opposite. By subtracting the Black Hat transform from the Top Hat transform and adding it back to the original image, we obtain an enhanced image that highlights the neurons in each image. The resulting transformed image provides improved contrast for quantification of biomarkers (Figure 2).

### Stable diffusion

**Figure 3:**
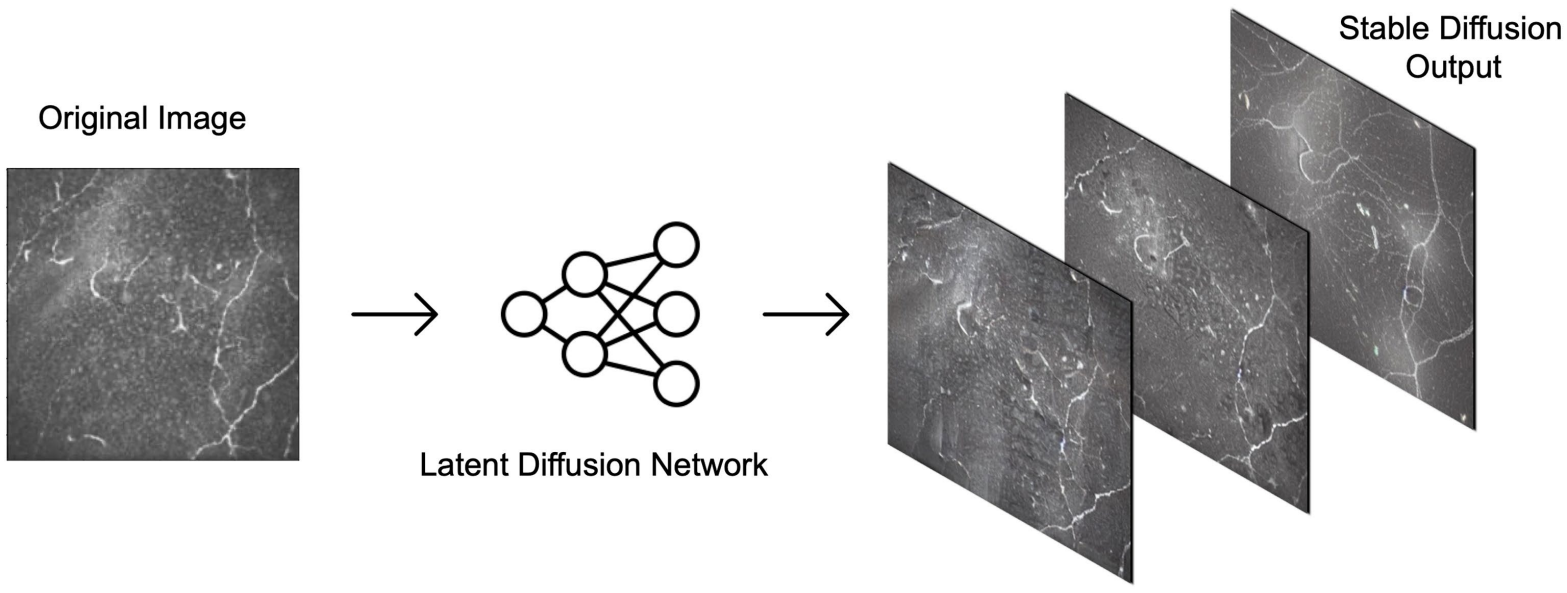
Examples of output possibilities from using stable diffusion on the confocal microscopy dataset. The latent diffusion network can be applied at various strengths to generate varying degrees of similarity between output images and the original image.

**Figure 4:**
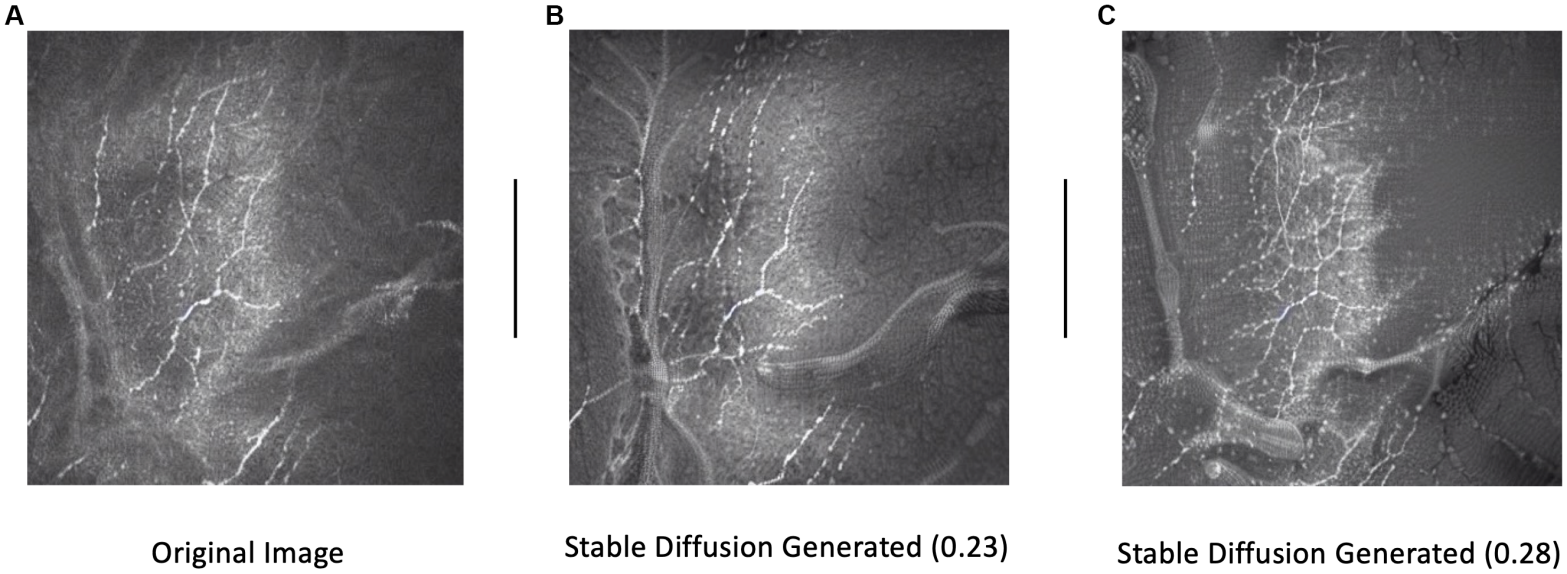
Example of an original image **(A)** and subsequent stable diffusion generated images at 0.23 **(B)** and 0.28 **(C)** strengths. Strength dictates the level of Gaussian noise infused into the original image.

Training set images were passed through Stable diffusion (SD), a model used to create additional artificial images by extending patterns. The model’s architecture, comprising a latent diffusion model (LDM) developed by the CompVis Group at Ludwig Maximilian University of Munich, consisted of two main components: (1) a variational autoencoder (VAE), a machine learning model that can infer and create new data relationships within images, and (2) a U-Net, a CNN designed for biomedical image segmentation by analyzing the image at different scales (15). Each image from our original dataset underwent 16 augmentation runs, with varying strengths of Gaussian noise infusion ranging from 0.2 to 0.28 (Figure 3). An example of the different strength hyper-parameter outputs can be seen in Figure 4.

At each iteration, the latent diffusion model generated new images by iteratively denoising random noise, guided by the CLIP text encoder pretrained on relevant concepts (19). This process allowed the generation of diverse representations of images while maintaining their original disease severity. The augmented images created through SD were then interlayed with our original training dataset before performing normalization, transformation, and finally, model training.

### Data Split and normalization

Image arrays and their corresponding metric values were read in after the image data was split into training and validation in a proportion of 70/30 by patient for model development. Separate lists were created to store the image features and the corresponding target variables for severity, fiber number, branch, fiber length, and tortuosity. These target variables represented the labels and metrics used for the subsequent analysis. Before feeding the image data into the model, normalization was applied to ensure consistent and standardized input. The image features were normalized to the range of [0, 1]. This normalization step ensured that all images had consistent intensity ranges and facilitated the convergence and stability of the model during training. Images were lastly feature scaled to ensure all input features have a similar scale or range.

### Model architecture and training

**Figure 5:**
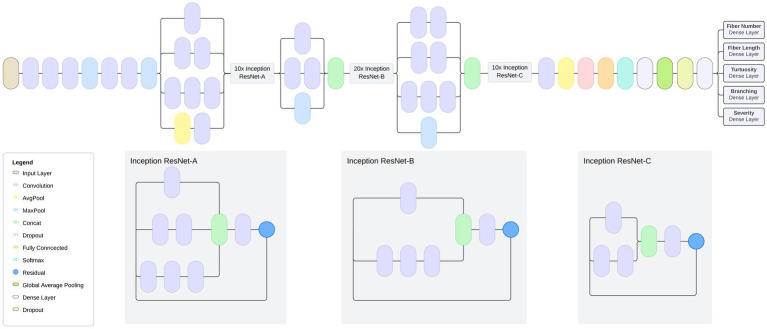
Model Architecture: Multi-task model based on InceptionResNetV2 transfer learning and U-Net architecture. Model is composed of a global average pooling layer followed by two fully connected layers. The model has a total of 54,764,136 parameters, of which 427,400 were trainable.

Machine learning models are often constructed on an existing model architecture, pre-trained on an existing dataset, and applied to a domain-specific task. A model architecture consists of a stack of layers that take an input image, perform a series of transformations on the image, and output a prediction based on the features learned from the image. The model architecture designed and tested in this study is based on InceptionResNetV2, a model pre-trained on the ImageNet dataset (20, 21), which provides a solid foundation for image feature extraction (Figure 5). A CNN is a machine learning model that feeds images through a series of convolutions to understand features within images. The InceptionResNetV2 model is a state-of-the-art CNN with residual connections that feed information to later layers of the model to aid with model training. To adapt the InceptionResNetV2 for our specific task, we appended it with additional layers, creating a Residual U-Net (ResUNet) architecture. The model was trained to predict multiple nerve morphology metrics and was composed of several key elements. First, residual blocks (a sequence of layers that take the output of a layer and add it to another layer) are integrated into the architecture, featuring skip connections that facilitate gradient flow and promote the effective extraction of both low-level and high-level features from the input images. These residual connections allow the model to bypass certain layers during training, enabling efficient network propagation and reducing the vanishing gradient problem, which is an issue where learned features are lost during model training.

Encoders and decoders are key components of a machine learning model architecture that break down an image to learn features and subsequently output these learned features to make a prediction. The model contains a decoder architecture which plays a critical role in reconstructing feature maps to their original spatial dimensions, allowing for image information to be more easily analyzed. This reconstruction is achieved through the incorporation of upsampling layers, which facilitate the restoration of high-resolution spatial details lost during the downsampling process in the encoder portion of the model. The upsampling layers work by replicating existing feature values to upscale the feature maps, effectively enlarging them to match their original dimensions. The decoder is composed of residual blocks, which leverage skip connections (connections that skip layers to deliver information to layers further within the architecture) to preserve essential feature information while upscaling the feature maps. Each residual block consists of two convolutional blocks with activation functions and batch normalization, which act as information processing layers. The skip connections within the residual blocks allow the decoder to directly access the original input features, facilitating the propagation of gradients and preventing the degradation of feature information during upsampling.

To enable the model to predict multiple metrics, we incorporated separate output branches for each target metric, namely severity, fiber number, branch characteristics, fiber length, and tortuosity. These output branches consist of convolutional layers with exponential linear unit (ELU) activation functions and L2 regularization. Activation functions allow predictions by applying a mathematical transformation to a model’s predicted output. L2 regularization is applied to calibrate the model during training by adjusting loss term values. To further optimize model training, we compiled it with the Adam optimizer using a learning rate of 0.001. Our choice of mean squared error (MSE) loss functions tailored for each output branch allowed us to effectively quantify the discrepancy between the predicted values and the ground truth. Additionally, we included mean absolute error (MAE) and root mean squared error (RMSE) as evaluation metrics to comprehensively assess the model’s performance during training and validation.

During model training there is a possibility for a model to overfit, where the model cannot generalize to data outside of the training set, leading to poor predictions. Augmentations of the images in the training set can be performed to help counter a model from overfitting to the training set. To enhance the model’s generalizability and prevent overfitting, we applied rigorous data augmentation techniques aside from and in combination with SD-generated images during training. We augmented the training images with rotations, translations, shearing, zooming, and horizontal flipping. Furthermore, we implemented a learning rate scheduler during training to dynamically adjust the learning rate, which is a mathematical value that determines the rate at which a model learns and processes image features, based on the validation loss. The scheduler reduced the learning rate by a factor of 0.1 if the validation loss did not improve after a certain number of epochs (the number of times that a machine learning model is trained), thus facilitating the model’s exploration of different areas in the loss landscape and preventing it from getting stuck in local minima. The final model was trained for 10 epochs, determined empirically on the training set, and tested on our held-out test set.

### Evaluation

The model’s performance was evaluated using root mean squared error (RMSE) and coefficient of determination (R^2^) as metrics for predicting fiber number, branch, fiber length, and tortuosity parameters. RMSE was employed to assess the accuracy of the model by quantifying the average difference between predicted values and ground truth from the validation dataset. R^2^ reported the proportion of variance in the prediction for each metric that was explained by the model. To evaluate the model’s predictive performance for severity, Area Under the Receiver Operating Characteristic Curve (AUC) was used to measure the ability of the model to distinguish between true positives and true negatives, and F1 score was used as a metric of the balance between performance and recall.

## Results

The results of the predictive models for various metrics related to limbal stem cell deficiency are summarized in Table 1.

**Table 1:** Multi-task neuro-morphology results: root mean squared error and R-squared values for stable diffusion trained model and model trained on solely original images.

Models	Fiber number RMSE	Fiber number R^2^	Branch RMSE	Branch R^2^	Fiber length RMSE	Fiber length R^2^	Tortuosity RMSE	Tortuosity R^2^
Stable diffusion + Original images	**4.44**	**0.63**	**4.54**	**0.63**	**5.56**	**0.80**	11.33	0.03
Original images only	5.04	0.53	5.13	0.52	6.3	0.74	**4.31**	**0.13**

Bold values represent the model with the best performance for each metric.

**Figure 6:**
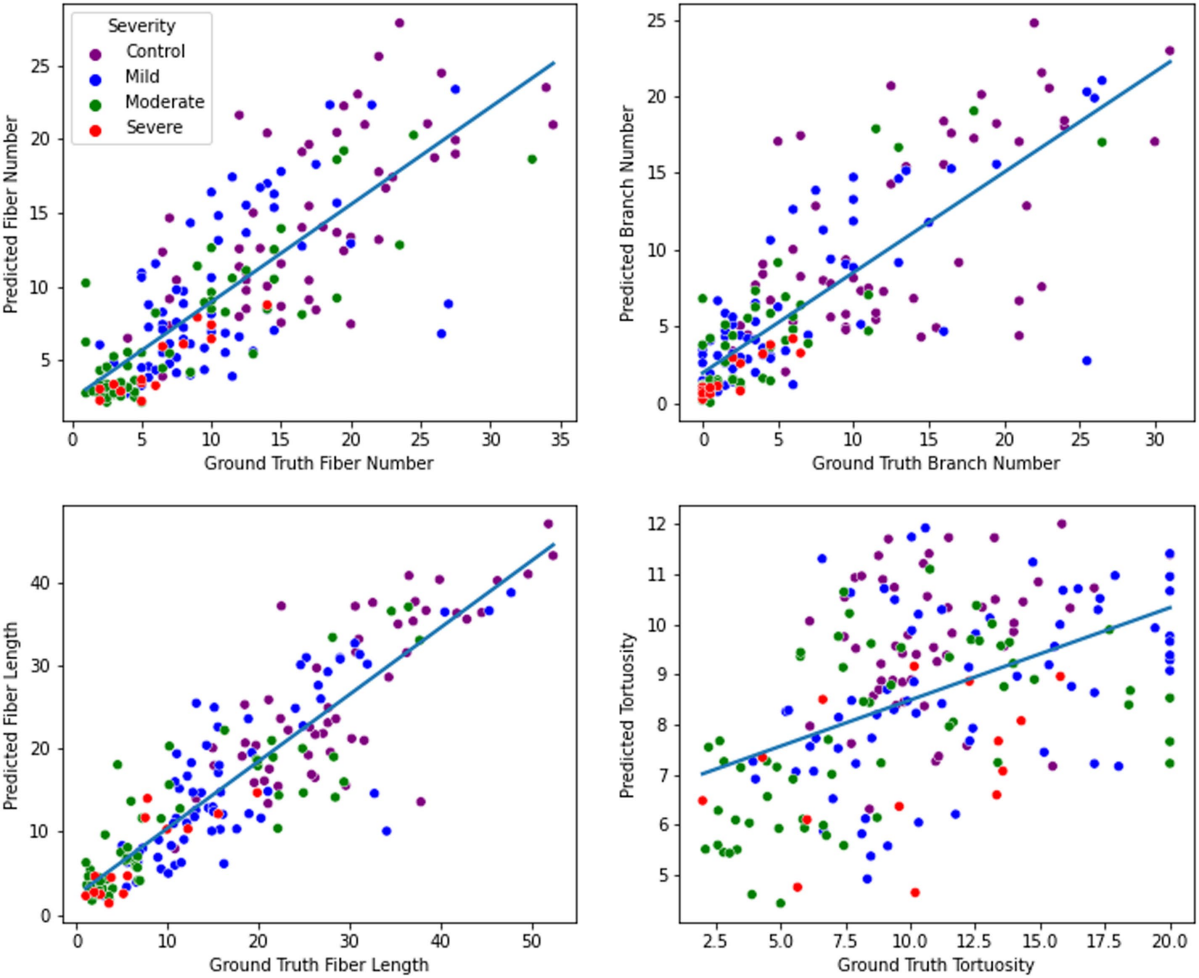
Nerve feature prediction performance. From top left to bottom right: fiber number, branch number, fiber length, and tortuosity. For each metric, a regression line was fitted to illustrate the direction and magnitude of the correlation between ground truth and predicted values.

When training the ResUNet on a combination of SD-augmented images and original images, the predictive model exhibited a notable performance increase across all morphology metrics aside from tortuosity when compared to the model trained solely on original images. For fiber number prediction, the model achieved an RMSE of 4.44 and an R^2^ of 0.63. Similarly, for branch prediction, the model obtained an RMSE of 4.54 with an R^2^ of 0.63. Most notably, the model demonstrated high accuracy in predicting fiber length, with an RMSE of 5.56 and a high R^2^ of 0.80. However, in the case of tortuosity prediction, the model’s performance showed room for improvement, yielding an RMSE of 11.33 and a relatively low R^2^ of 0.03. Distribution of predictions and ground truth values can be found in Figure 6.

In contrast, when training the model on only original images as input, the model’s predictive capabilities were noticeably lower compared to the SD-augmented model. The RMSE for fiber number was 5.04, and the corresponding R^2^ was 0.53, indicating a moderate level of accuracy. For branch prediction, the model obtained an RMSE of 5.13 and an R^2^ of 0.52. For fiber length estimation, the model achieved an RMSE of 6.3 and an R^2^ of 0.74. Finally, regarding tortuosity prediction, the model showed an RMSE of 4.31 and an R^2^ of 0.13.

Disease severity was assessed individually in a single metric variation of the residual U-net model. This single task version of the model was also trained on the SD and non-SD supplemented data sets. Disease severity was stratified and assessed as disease vs. no disease (control), control/mild vs. moderate/severe, and severe vs. non-severe. Performance was compared against a classifier based on the morphological features that were assessed manually during routine clinical practice. A summary of disease severity statistics can be found in Table 2.

**Table 2:** Severity comparisons and corresponding AUC, Precision, Recall, F1, and accuracy values with and without stable diffusion (SD) images and using manually assessed metrics only.

		AUC	Precision	Recall	F1	Accuracy
Control vs. mild, moderate, severe	Manual	0.857	**0.980**	0.683	0.805	0.758
	SD	0.855	0.947	**0.754**	**0.839**	**0.789**
	No SD	**0.867**	0.970	0.676	0.797	0.747
Control, mild vs. moderate, severe	Manual	0.803	0.718	0.689	0.703	**0.778**
	SD	0.810	**0.838**	0.667	**0.743**	**0.778**
	No SD	**0.816**	0.653	**0.838**	0.734	0.768
Control, mild, moderate vs. severe	Manual	0.733	0.126	0.929	0.222	0.531
	SD	**0.765**	**0.146**	**1.00**	**0.255**	**0.577**
	No SD	0.746	0.130	**1.00**	0.230	0.515

Bold values represent the model with the best performance for each metric in each severity classification.

**Figure 7:**
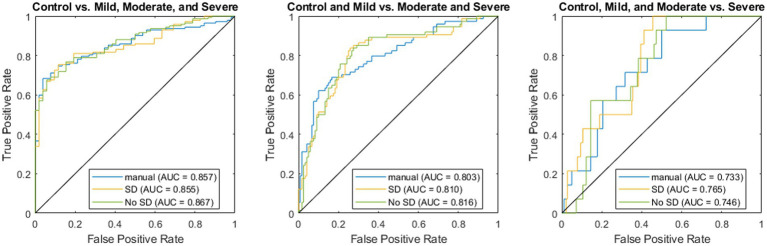
Receiver operator characteristic curves for regression using manually derived features (blue), deep learning using stable diffusion (SD; orange), and deep learning without SD (green).

In all three scenarios, the ResUnet model with SD outperformed classification using manual morphological metrics in terms of F1 (0.839 vs. 0.805, 0.743 vs. 0.703, and 0.255 vs. 0.222, respectively). The accuracy using the ResUnet with SD produced higher accuracy than the manual metrics for classifying the controls and severe cases (0.789 vs. 0.758 and 0.577 vs. 0.531, respectively), and they had the same accuracy when classifying between mild and moderate cases (0.778). The Area Under the Receiver Operator Characteristic Curve (AUC) for the ResUnet with SD was higher in the moderate and severe cases (0.810 vs. 0.803 and 0.765 vs. 0.733, respectively), but slightly lower in the control classification (0.855 vs. 0.857; Figure 7).

When comparing the classification with and without SD in the classifier, the model with SD again had higher accuracy (0.789 vs. 0.747, 0.778 vs. 0.768, and 0.577 vs. 0.515) and F1 (0.839 vs. 0.797, 0.743 vs. 0.734, and 0.255 vs. 0.230). The model without SD had higher AUC values for the control (0.855 vs. 0.867) and mild classifications (0.810 vs. 0.816), while the SD model had the highest AUC when classifying the severe cases (0.765 vs. 0.746).

## Discussion

This study presents a novel approach to enhancing the performance of multi-task nerve morphology prediction by incorporating images generated through latent diffusion models as a form of training set bolstering. Specifically, SD-generated images were introduced as an augmentation technique to complement the original dataset in training the residual U-Net architecture. The results demonstrate the potential of this approach, as the inclusion of SD-generated images led to a notable improvement in the model’s nerve morphology prediction performance when compared to the same model trained solely on original images. The introduction of SD-generated images allowed the residual U-net model we created to leverage additional synthetic samples, resulting in enhanced generalization and predictive capabilities. It is important however to acknowledge the inherent limitations of training machine learning models on generated data.

Previous work on using nerve features for severity prediction has theorized that morphological changes to nerves become more pronounced as disease severity increases, which is supported by the results shown (22). The high AUC of 0.855 supports the claim that nerve morphology can be used as an effective predictor of LSCD severity. In a larger pipeline, the disease severity prediction by nerve morphology can be supplemented by other predictors such as cell morphology and basal cell density. While in the context of this study nerve density was a sufficient predictor, some confocal microscopy images contain less pronounced nerve morphology. Thus, the predictions shown would likely be most beneficial as part of a larger pipeline that examines multiple features of corneal images for disease severity prediction.

Overall, the inclusion of SD-augmented images in the ResUnet model led to substantial improvements in predicting fiber number, branch, and fiber length metrics associated with limbal stem cell deficiency. The enhanced predictive accuracy demonstrated the model’s capacity to better capture intricate spatial features and relationships, resulting in more reliable and robust estimations. The models had more difficulty learning tortuosity; this difficulty is understandable as the clinical evaluation can be highly variable in images with few nerves present (i.e., severe cases). While certain challenges remain in predicting tortuosity accurately, the promising outcomes highlight the potential of the proposed approach for comprehensive and precise assessments of limbal stem cell deficiency metrics.

The inclusion of SD training images was less helpful in classifying severity than individual morphological features. While the SD model had consistently higher accuracy and F1, the differences were relatively small and largely resulted from a different classification threshold as demonstrated by the similar ROC curves (Figure 7). The highest difference between the two was in classifying severe cases, which had the fewest training examples, which could indicate that SD helped overcome this lack of data. The overall similarity in predictions could be a result of the classification using nerve images alone whereas clinical diagnosis utilizes multiple other data sources and modalities. The utility of these models is demonstrated in the fact that the deep learning model outperforms a classifier trained only on the metrics manually assessed from these images through standard clinical practice. Future directions can extend these methods to incorporate these other data sources to create a more holistic classifier that better represents the full spectrum of information available to a clinician.

SD images may be beneficial for training the model in terms of increasing the dataset size, although it potentially may limit the features that can be learned. SD inherently alters the structure of an image, although the degree to which this affects the model’s ability to learn features is unknown. The addition of SD images in the training dataset may improve model predictions as the feature identification task becomes more difficult. Conversely, the inclusion of SD images may limit the model performance by forcing the model to learn extraneous features that were created as a result of including the SD-generated images.

Despite the limitations of SD, our findings highlight the potential benefits of incorporating latent diffusion-generated images as a means of data augmentation in the context of nerve morphology regression. The approach not only offers an avenue to expand the training dataset but also provides an opportunity to explore diverse representations of the same images while preserving their semantic meaning. Further investigations into mitigating overfitting and optimizing the augmentation process are warranted to unlock the full potential of this novel technique. The combination of real and generated data may lead to more robust and accurate predictive models when faced with limited training data. This can serve to facilitate advancements in the diagnosis and treatment of nerve-related pathologies and beyond.

### Future directions

In the future, additional processing methods such as bootstrapping, creating larger SD image sets, and modifying the noise parameters and weights in our latent diffusion model can be performed to improve results and further validate efficacy of the model. Additionally, it is possible that employing SD images changes the patterns or number of neurons in an image, which could make it more representative of a different disease state from the original image. Thus, including a step that segments the neurons in both the raw and SD images, compares the pixel volume of each, and assigns a weight to the SD image based on the difference when compared to its unaltered counterpart would help to correct this issue. Alternatively, once a model is trained to be proficiently accurate on raw data, it could be used to assign predicted metric values on SD images. Further research is required to validate the use case for including SD images in training data as a method of data augmentation.

## Conclusion

This study demonstrates the effectiveness of incorporating SD-generated images as an augmentation technique to enhance the performance of a multi-task nerve morphology prediction model for limbal stem cell deficiency (LSCD). The inclusion of SD images significantly improved the model’s predictive capabilities for fiber number, branch, and fiber length metrics associated with LSCD, showcasing its potential for precise assessments of this disorder. However, challenges remain in accurately predicting tortuosity and disease severity, warranting further investigation. While this approach shows promise in leveraging synthetic data to bolster training sets, careful consideration of overfitting and model convergence is essential. By refining the preprocessing methods and exploring additional augmentation techniques, this novel approach may lead to more robust and accurate predictive models for nerve morphology analysis and disease severity prediction, potentially improving clinical workflows in the future.

## Data availability statement

The raw data supporting the conclusions of this article will be made available by the authors, without undue reservation.

## Ethics statement

The studies involving humans were approved by UCLA Institutional Review Board. The studies were conducted in accordance with the local legislation and institutional requirements. The participants provided their written informed consent to participate in this study.

## Author contributions

DG: Investigation, Methodology, Software, Writing – original draft, Writing – review & editing. TT: Investigation, Software, Writing – original draft, Writing – review & editing. VR: Investigation, Software, Writing – original draft, Writing – review & editing. CB: Data curation, Writing – review & editing. NS: Formal Analysis, Writing – review & editing. MV: Formal Analysis, Writing – review & editing. TS-B: Data curation, Writing – review & editing. CA: Methodology, Resources, Supervision, Writing – review & editing. SD: Conceptualization, Funding acquisition, Resources, Writing – review & editing. WS: Conceptualization, Data curation, Formal Analysis, Project administration, Supervision, Writing – review & editing.
